# Doping Induced Structural Stability and Electronic Properties of GaN Nanotubes

**DOI:** 10.1155/2014/984591

**Published:** 2014-02-24

**Authors:** Anurag Srivastava, Mohammad Irfan Khan, Neha Tyagi, Purnima Swaroop Khare

**Affiliations:** ^1^Advanced Materials Research Group, Computational Nano Science & Technology Lab, ABV-Indian Institute of Information Technology & Management Gwalior (M.P.) 474015, India; ^2^School of Nanotechnology, Rajiv Gandhi Proudyogiki Vishwavidyalaya Bhopal (M.P.) 462033, India

## Abstract

The present paper discusses the effect of manganese doping on the structural stability and electronic band gap of chiral (2, 1), armchair (3, 3), and zigzag ((6, 0) and (10, 0)) single walled GaN nanotube by using density functional theory based Atomistix Toolkit (ATK) Virtual NanoLab (VNL). The structural stability has been analyzed in terms of minimum ground state total energy, binding, and formation energy. As an effect of Mn doping (1–4 atoms), all the GaN nanotubes taken into consideration show semiconducting to metallic transition first and after certain level of Mn doping changes its trend.

## 1. Introduction

The study of carbon nanotubes from last few decades has grown widely. The first step for growth of carbon nanotubes was fullerene structure, which was discovered in 1985. In 1991 Iijima [1] synthesizes multiwall carbon nanotubes by Arc Discharge Method, in 1993 Bethune et al. [2] reports the synthesis of single wall carbon nanotube using cobalt as a catalyst. Carbon nanotubes become more interesting because of their various properties, due to the changing the diameter and helical arrangement of carbon nanotubes, it shows metallic as well as semiconducting behavior [3, 4]. Carbon nanotubes are basically a rolled one atom thick layer of sheet called Graphene, where the length of tube is larger than the tube diameter [3]. Carbon nanotubes show a wide range of applications in the area of electronic, optical, medical, mechanical, and other industries [5–11]. Due to the advanced application of carbon nanotubes, researchers are also trying for other materials nanotubes. GaN [12] is of great importance due to its high thermal, mechanical stability, and optoelectronic properties. Due to the large direct band gap of GaN, it is used in high-temperature, high-power electronics and short wavelength (UV) detectors. Few studies [13, 14] are devoted to the stability analysis of GaN nanotube, where GaN nanotubes are found to be stable and can be synthesized under some extreme conditions. Literature also reports that the energy gap decreases by decreasing the tube diameter and zigzag GaN nanotubes show direct band gap, whereas armchair and chiral GaN nanotubes have the indirect band gap. Our group has also investigated the structural and electronic properties of several nanotubes [15–19]. Mg-doped [20, 21] GaN nanocages and nanotubes can be magnetic with Mg-contributed spins distributed over the neighboring N sites. The study [22] based on Cr-doped GaN nanotubes confirms the coupling between two Cr atoms mediated by the neighboring N is ferromagnetic, but changes to ferrimagnetic as the cluster grows. Literature [23] shows the effect of nitrogen doping on the electronic and magnetic properties of carbon nanotubes, where, at small N-doping, the finite-length zigzag CNTs maintains an ant ferromagnetic (AFM) ground state, whereas the conversion from AFM to a nonmagnetic state occurs at high N-concentrations. Researchers [24–26] have also analyzed the properties of III-nitride nanotubes under compression, where, the Young's modulus shows the spin suppression independent of tube diameter.

## 2. Computational Detail

The present theoretical study has been made by using abinitio approach based on density functional theory (DFT) [27, 28] for the analysis of electronic properties of GaN nanotubes, we used Atomistix Toolkit-Virtual NanoLab (ATK-VNL) [29] tool which uses linear combination of atomic orbital's (LCAOs) with norm conserving pseudo potential. Here GGA approximation is used as exchange correlation function in the form of revised Perdew, Burke, and Ernzerhof (PBE) [28] type parameterization. For describing the valence electron, double-*ζ* double polarized basis set has been used. To achieve the convergence, mesh cutoff energy has been finalized on the ground of convergence principle and for the present computation 150 Ryd has been predicted as the most suitable one after several convergence test, throughout the calculation. The minimization of total energy has been performed to optimize the bond length of the nanotubes. In the entire set of optimization run, maximum force tolerance was set at 0.05 eV/Å. For achieving the total energy convergence we have used K-point sampling of 1 × 1 × 20.

## 3. Results and Discussion

**Figure 1:**
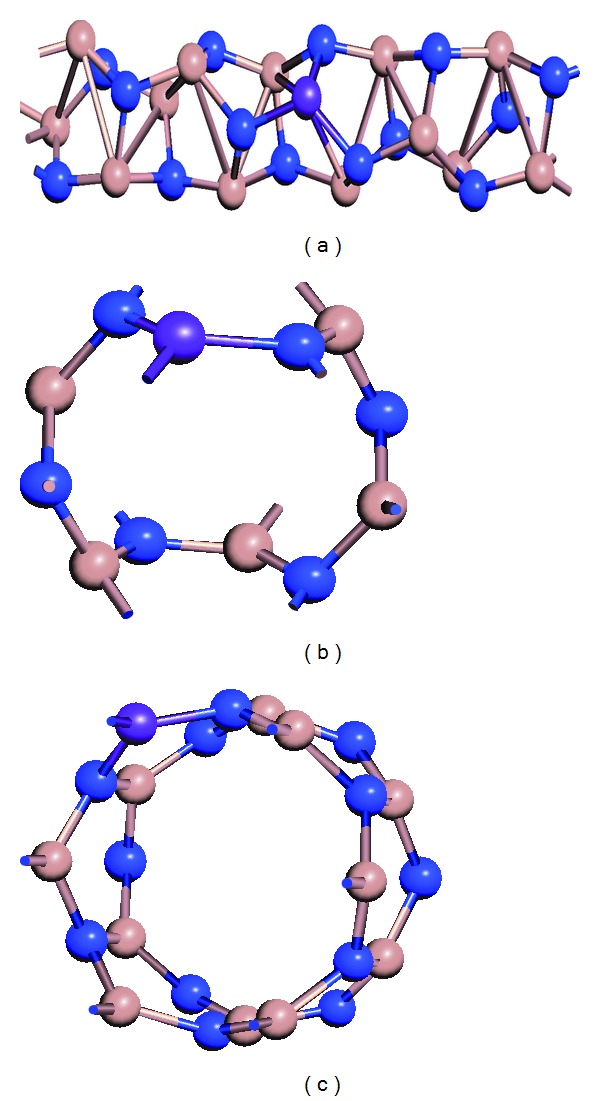
Mn doped GaN nanotubes of different tube indices: (a) chiral (2, 1), (b) armchair (3, 3), and (c) zigzag (6, 0). Blue, brown, and purple colors correspond to nitrogen gallium and manganese atoms, respectively.

In the present work, we have used (2, 1) chiral, (3, 3) armchair, and (6, 0) and (10, 0) zigzag single walled GaN nanotubes of diameter 2.83 Å, 5.30 Å, 6.08 Å, and 10.01 Å, respectively. Initially the structural stability and electronic properties of undoped (2, 1) chiral, (3, 3) armchair, (6, 0) and (10, 0) zigzag GaN nanotubes have been investigated. Further we have seen the effect of manganese doping on GaN nanotubes structural stability and electronic properties in the form of replacing the Ga atoms with the manganese (Mn) atoms up to four; Figure 1 shows the one manganese doped GaN nanotubes of different chirality.

## 4. Structural Stability

**Figure 2:**
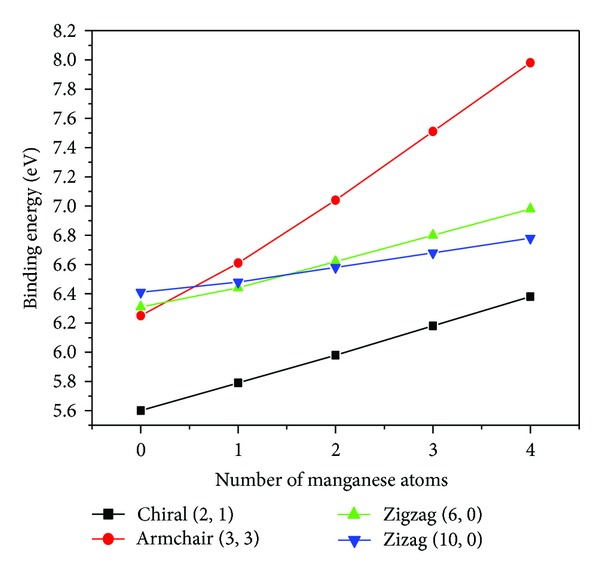
Binding energy as a function of Mn atoms, where black square, red circle, green upper triangle, and blue below faced triangle correspond to chiral (2, 1), armchair (3, 3), (6, 0), and (10, 0), and zigzag GaN nanotubes, respectively.

The structural stability analysis of (2, 1) chiral, (3, 3) armchair, and (6, 0) and (10, 0) Zigzag single walled GaN nanotubes has been performed by minimizing the total energy of ground state as a function of bond length. For the nanostructures the stability can be best defined through the binding/cohesive energy for the same. We have calculated the binding as well as formation energy of these nanotubes with the help of total energy of ground state tabulated in Table 1. The ground state total energy of GaN nanotubes shows a decreasing trend with the manganese concentration, whereas, the binding energy increases and hence the tubes are getting more and more stable stable with higher Mn concentration. The bond length of doped nanotubes is also decreasing with increases in the concentration of manganese atoms in all the GaN nanotubes taken into consideration. On the other hand the binding energy of manganese doped GaN nanotubes shows the proportional relation with the manganese atoms as shown in Figure 2. With the highest binding energy 4-Mn doped (3, 3) GaN shows the most stable structure amongst all others taken into consideration. The binding energy of the single wall GaN nanotubes has been obtained from the following relation:

(1) $$\begin{array}{c} E_{\text{Binding}}=-\frac{\left(E_{T}-xE_{\text{Ga}}-yE_{\text{N}}-zE_{\text{Mn}}\right)}{x+y+z}, \end{array}$$

where *E*_*T*_ is the total energy of single wall GaN nanotubes, *E*_Ga_, *E*_N_, and *E*_Mn_ are the total energy of single-free gallium, nitrogen, and manganese atoms and *x*, *y*, and *z* are the number of gallium, nitrogen, and manganese atoms in the supercell, respectively.

**Figure 3:**
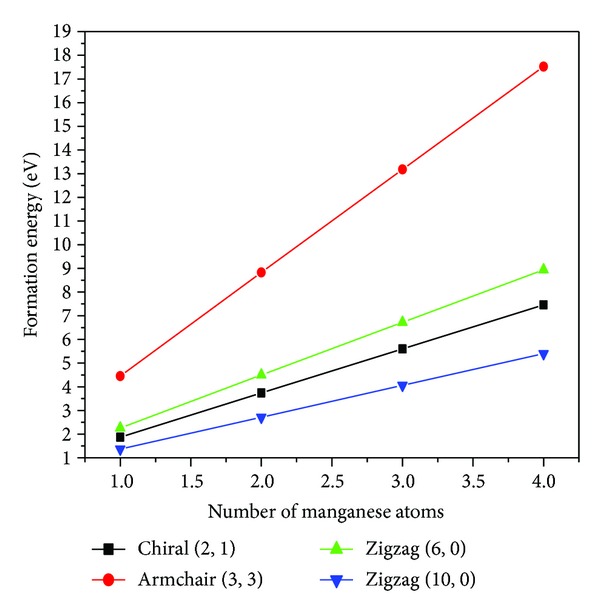
Formation energy as a function of Mn atoms, where black square, red circle, green upper triangle, and blue below faced triangle correspond to chiral (2, 1), armchair (3, 3), (6, 0), and (10, 0), and zigzag GaN nanotubes, respectively.

Moreover the formation energy of manganese doped GaN nanotube is calculated by using the ground state energy of relaxed structures. The formation energy of GaN nanotubes has been calculated by using the following relation:

(2) $$\begin{array}{c} E_{\text{Formation}}=\frac{\left(E_{\text{Doped}\;\text{GaNNT}}-E_{\text{Pristine}}-xE_{\text{Mn}}\right)}{N}, \end{array}$$

where *E*_Doped  GaNNT_ is the total energy of doped GaN nanotube in the supercell, *E*_Pristine_ is the total energy of pristine GaN nanotube, *E*_Mn_ is the total energy of free manganese atom, and *x* and *N* are the number of manganese atoms and the total number of atoms in the supercell, respectively. The calculated formation energies of all the nanotubes taken into consideration are given in Table 1.  Figure 3 shows the formation energy plot as a function of manganese doped GaN nanotubes, and analysis shows that by increasing the number of manganese atoms in GaN nanotubes the formation energy also increases and reaches towards more stability.

### 4.1. Electronic Properties

The electronic properties (band structure and density of states) of pristine and manganese doped GaN nanotubes have been computed at the stabilized bond length. The obtained results give the information on the band gap, its nature, and probability of finding the electron near the Fermi level. The calculated band gaps of all the GaN nanotubes taken into consideration are reported in Table 1 and discussed in the following sections.

### 4.2. Chiral (2, 1) GaN Nanotubes

**Figure 4:**
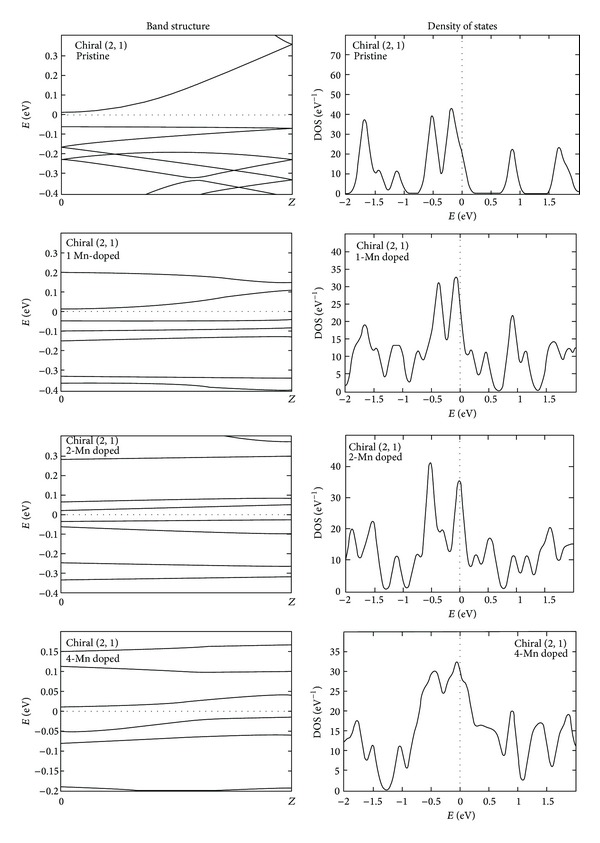
The band structure and density of states of pristine and Mn doped chiral (2, 1) GaN nanotubes.

The band structure plot of chiral (2, 1) type pristine GaN nanotube shows a very small indirect band gap of about 0.072 eV, shown in Figure 4. On increasing the Mn concentration in the GaN nanotubes the valance and conduction band comes near to the Fermi level and shows the smallest band gap of about 0.027 eV at 4 Mn doped GaN nanotube, whereas at the 3 Mn doped tube it is metallic. The DOS profile of chiral GaN nanotubes is shown in Figure 4, where in case of pristine chiral (2, 1) GaN nanotube the highest peak in the conduction band region is present at around 16 eV and the highest peaks in the valance band region are at around −13.25 eV along with few disrupted peaks. At 1 Mn doped GaN nanotubes the DOS profile having the small disrupted peaks near the Fermi level and the highest peak in the conduction band region is present at around 12.4 eV and in the valance band region at −13.25 eV. The DOS profile confirms that on increasing the Mn concentration in GaN nanotubes, the presence of electrons near the Fermi level increases.

### 4.3. Armchair (3, 3) GaN Nanotubes

**Figure 5:**
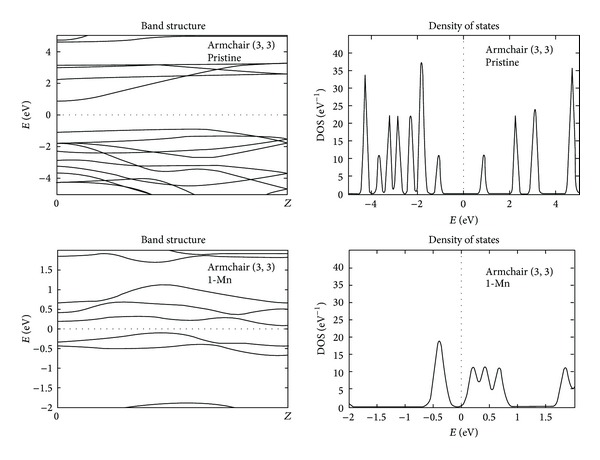
The band structure and density of states profile of pristine and Mn doped armchair (3, 3) GaN nanotubes.

The band structure plot of pristine armchair (3, 3) GaN nanotube shows an indirect band gap of about 1.75 eV, shown in Figure 5. Here too, the introduction of Mn at the site of Ga in GaN nanotube brings the conduction band near to the Fermi level and for single Mn doping the gap reduces to 0.19 eV. On increasing the Mn concentration in the GaN nanotubes the energy levels of valance and conduction band cross the Fermi level and transform the semiconducting behavior of these nanotubes into metallic. To understand the localization of electrons near the Fermi level, we have also plotted the density of states profile of these nanotubes. The DOS profile of armchair (3, 3) GaN nanotubes is shown in Figure 5, where, in case of pristine armchair (3, 3) GaN nanotube, the highest peak in the conduction band region can be seen at around 4.7 eV and a highest peak in the valance band region at around −1.8 eV along with few disrupted peaks. At 1 Mn doped GaN nanotubes, the DOS profile having few disrupted peaks near the Fermi level and the highest peak in the conduction band region is present at around 9.5 eV and in the valance band region at around −3 eV. The DOS profile shows that, by increasing the concentration of Mn atom in GaN nanotubes, the localization of electron increases near the Fermi level.

### 4.4. Zigzag (6, 0) and (10, 0) GaN Nanotubes

**Figure 6:**
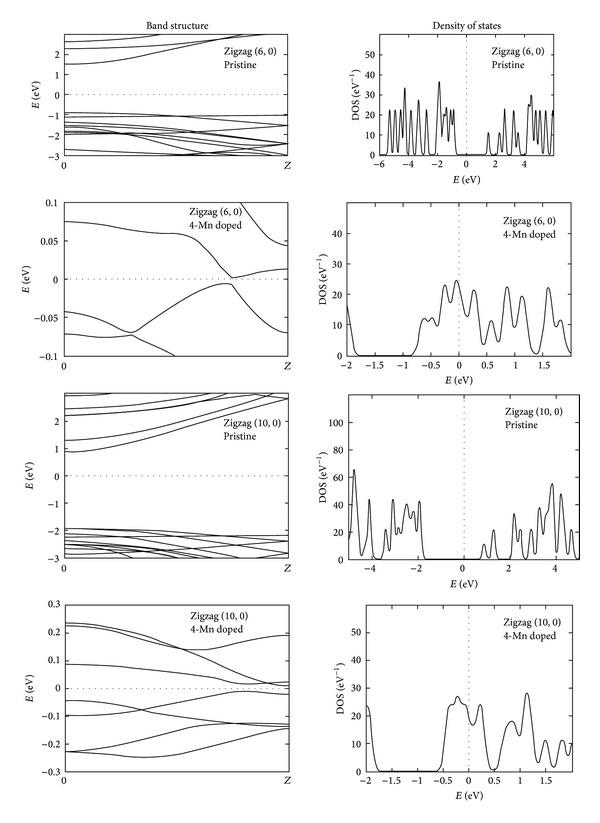
The band structure and density of states profile of pristine and Mn doped zigzag (6, 0) and (10, 0) GaN nanotubes.

The (6, 0) and (10, 0) zigzag pristine and three manganese doped GaN nanotubes show direct band gap of about 2.39 and 2.80 eV, respectively. The nanotube transforms to metallic by increasing the number of manganese atoms from one to four in (6, 0) and (10, 0) GaN nanotubes, while with three manganese doping they show a very small direct band gap of about 0.0083 eV and 0.0018 eV, respectively. The band structure and DOS profile of zigzag (6, 0) and (10, 0) GaN nanotubes are shown in Figure 6, where in case of pristine zigzag (6, 0) GaN nanotube the highest peak in the conduction band region is present at around 12.4 eV and a highest peak in the valance band region at around −13.5 eV, with few disrupted peaks. At 1 Mn doped zigzag (6, 0) and (10, 0) GaN nanotubes the DOS profile having the small peaks near the Fermi level and the highest peak in the conduction band region is present at around 7.4 eV and 2.75 eV and in the valance band region at −15 eV and −5 eV, respectively. The DOS profile shows that, by increasing the Mn concentration in GaN nanotubes, the number of peaks near the Fermi level increases in these zigzag (6, 0) and (10, 0) GaN nanotubes.

## 5. Conclusion

Ab-initio analysis has been performed for determining the structural stability and electronic properties of single walled Mn doped GaN nanotubes. With the highest binding energy 4 Mn doped (3, 3) GaN nanotube have been defended as the most stable structure amongst all others taken into consideration. The doping of manganese in pristine GaN nanotube decreases the band gap up to certain level and again changes its trend. The findings reported in the present paper on the doping effect will certainly provide a new dimension to the use of these Mn doped GaN nanotubes in a variety of applications.

## Figures and Tables

**Table 1:** The bond length, binding energy, formation energy, and bandgaps of different types of doped and undoped single walled GaN nanotubes.

Nanotubes	Bond length (Å)	Total energy (eV)	Binding energy (eV)	Formation energy (eV)	Band gap (eV)
Pristine chiral (2, 1)	1.94	−4650	5.60	—	0.072I
One-Mn atom doping	1.94	−5206.36	5.79	1.87	0.056I
Two-Mn atom doping	1.93	−5762.87	5.98	3.74	0.045I
Three-Mn atom doping	1.93	−6319.66	6.18	5.60	M
Four-Mn atom doping	1.92	−6876.35	6.38	7.46	0.027I
Pristine armchair (3, 3)	1.85	−2000.69	6.25	—	1.75I
One-Mn atom doping	1.84	−2553.10	6.61	4.45	0.19I
Two-Mn atom doping	1.82	−3112.41	7.04	8.83	M
Three-Mn atom doping	1.80	−3669.09	7.51	13.18	M
Four-Mn atom doping	1.78	−4225.88	7.98	17.52	M
Pristine zigzag (6, 0)	1.84	−4002.60	6.31	—	2.39D
One-Mn atom doping	1.84	−4556.99	6.44	2.26	M
Two-Mn atom doping	1.83	−5112.42	6.62	4.50	M
Three-Mn atom doping	1.82	−5667.82	6.80	6.72	0.0083
Four-Mn atom doping	1.82	−6223.29	6.98	8.94	M
Pristine zigzag (10, 0)	1.82	−6675.13	6.41	—	2.80D
One-Mn atom doping	1.82	−7229.13	6.48	1.37	M
Two-Mn atom doping	1.82	−7784.24	6.58	2.71	M
Three-Mn atom doping	1.82	−8339.41	6.68	4.06	0.0018D
Four-Mn atom doping	1.81	−8894.57	6.78	5.40	M

D: direct, I: indirect, and M: metallic.
